# Using Selective Enzymes to Measure Noncanonical DNA Building Blocks: dUTP, 5-Methyl-dCTP, and 5-Hydroxymethyl-dCTP

**DOI:** 10.3390/biom13121801

**Published:** 2023-12-15

**Authors:** Éva Viola Surányi, Viktória Perey-Simon, Rita Hirmondó, Tamás Trombitás, Latifa Kazzazy, Máté Varga, Beáta G. Vértessy, Judit Tóth

**Affiliations:** 1 Institute of Enzymology, Research Centre for Natural Sciences, H-1117 Budapest, Hungary; perey.si.vi@gmail.com (V.P.-S.); hirmondo.rita@ttk.hu (R.H.);; 2 Department of Applied Biotechnology and Food Sciences, Budapest University of Technology and Economics, H-1111 Budapest, Hungary; 3 Department of Genetics, ELTE Eötvös Loránd University, H-1117 Budapest, Hungarymvarga@ttk.elte.hu (M.V.)

**Keywords:** dNTP incorporation, dNTP pool, nucleotide hydrolysis, nucleotide analogs

## Abstract

Cells maintain a fine-tuned balance of deoxyribonucleoside 5′-triphosphates (dNTPs), a crucial factor in preserving genomic integrity. Any alterations in the nucleotide pool’s composition or chemical modifications to nucleotides before their incorporation into DNA can lead to increased mutation frequency and DNA damage. In addition to the chemical modification of canonical dNTPs, the cellular de novo dNTP metabolism pathways also produce noncanonical dNTPs. To keep their levels low and prevent them from incorporating into the DNA, these noncanonical dNTPs are removed from the dNTP pool by sanitizing enzymes. In this study, we introduce innovative protocols for the high-throughput fluorescence-based quantification of dUTP, 5-methyl-dCTP, and 5-hydroxymethyl-dCTP. To distinguish between noncanonical dNTPs and their canonical counterparts, specific enzymes capable of hydrolyzing either the canonical or noncanonical dNTP analogs are employed. This approach provides a more precise understanding of the composition and noncanonical constituents of dNTP pools, facilitating a deeper comprehension of DNA metabolism and repair. It is also crucial for accurately interpreting mutational patterns generated through the next-generation sequencing of biological samples.

## 1. Introduction

The persistent demand to enhance our comprehension of cellular dNTP homeostasis has driven the development of new methods for precisely quantifying the composition of the cellular dNTP pools [1,2,3,4,5,6,7,8]. The appropriate concentration of dNTPs and the fine-tuned balance between them are essential for fundamental biological processes, including DNA synthesis [9], cell wall synthesis [10,11], antiviral defense [12], and antibody hypermutation [13]. Current dNTP quantification methods focus primarily on canonical dNTPs. However, even normal dNTP biosynthetic processes constantly produce a noncanonical dNTP, dUTP [14]. Furthermore, dNTPs are chemically vulnerable and sensitive to oxidation, alkylation, and deamination, leading to various noncanonical forms [15]. Since noncanonical dNTPs can also be incorporated into the DNA, resulting in mispairing or enzyme-induced lesions [15], their presence is a potential source of increased mutagenesis and DNA damage. Usually, the level of noncanonical dNTPs is kept low by the corresponding dNTP hydrolyzing enzymes in the cells. However, in some instances, an increase in noncanonical dNTP levels will result from enzyme dysfunctionality, drug treatment, oxidative or alkylation stress, viral infection, or other factors [16]. The so-called dNTP pool sanitizing enzymes are specialized for digesting noncanonical dNTPs. For example, the dUTPase enzyme specifically hydrolyzes dUTP [17], the MutT enzyme hydrolyzes 8-oxo-dGTP [18] or 5-methyl-dCTP [19], and the ITPase and MazG family enzymes hydrolyze dITP and 2-oxo-dATP [20,21], respectively. To obtain a more accurate understanding of the possible effects of changes in the dNTP pools, we propose to extend the accessible dNTP quantification capabilities for noncanonical dNTPs.

The current dNTP measurement methods can be divided into high-performance liquid chromatography (HPLC)-based methods and DNA-polymerization-based enzymatic assays. The HPLC can be coupled with a UV or mass-spectrometry (MS) detection. Although HPLC-UV and HPLC-MS are applicable to measure noncanonical dNTPs, both have disadvantages that narrow their usability. One of the most significant drawbacks of the HPLC-UV method is the need for a large sample size. Noncanonical dNTPs are expected to be less abundant than normal dNTPs, which may increase the demand for larger sample sizes. The HPLC-MS technique has high sensitivity and yields the best performance of all methods. However, the bottleneck of using this method is the need for specialized instrumentation and high expertise. Moreover, the separation of dNTPs is mainly carried out using an ion-pairing reagent, which is incompatible with other MS uses. Therefore, only dedicated equipment is used for dNTP measurements. We recently collected most of the published cellular dNTP results into a database, which revealed that only 3.7% of the measured dNTP data originate from HPLC-MS measurements [22]. This observation suggests that, despite its high performance, this method may not be readily available to numerous laboratories that are interested in measuring dNTP pools. The HPLC-UV technique is used in 28% of the cases, while the most popular method for measuring dNTPs is the enzymatic dNTP incorporation method, with a share of 63% in published dNTP data. dNTP incorporation methods are based on extending a DNA primer annealed to a template limited by the amount of the specific dNTP to be measured. For the detection of the polymerization rate or extent, previously developed methods used radioactive labels either on one of the non-limiting dNTPs or the primer DNA [5,23,24]. Radioactivity-based detection techniques used to be the most widespread in the scientific community [22]; however, radioactive methods pose a health and environmental risk and require special handling. Moreover, these methods are time-consuming and labor-intensive. Therefore, they cannot be applied to high-throughput investigations. Although the radioactive dNTP incorporation method enables the precise quantification of dUTP [25], the abovementioned drawbacks limit its applicability nowadays. Recently, many dNTP incorporation methods have been developed using fluorescence detection [1,2,3,4,7,26]. Its widespread availability and adaptability for a medium-to-high throughput implementation render the dNTP incorporation method with fluorescent detection a favorable choice to extend its measurement capability with noncanonical dNTPs.

In this work, we aimed to establish methods for quantifying dUTP, 5-methyl-dCTP, and 5-hydroxymethyl-dCTP. Therefore, we developed protocols using house-cleaning enzymes to selectively eliminate the canonical or the noncanonical dNTP species from the dNTP mixture. The two significant advances that made these quantifications feasible are (i) the modification of the Purhonen et al. assay [2] with the use of the Q5U enzyme and the addition of the dUTPase enzyme [27,28], and (ii) the application of the mycobacterial Dcd:dut enzyme to selectively eliminate dCTP while leaving the noncanonical dCTP derivatives intact.

## 2. Methods

### 2.1. Mycobacterial Strains and Growth Conditions

The *Mycobacterium smegmatis* mc^2^155 strain used for the experiments was grown in Lemco liquid culture (5 g/L Lemco, 5 g/L NaCl, 10 g/L Bacto peptone, 0.05% Tween-80) or on solid Lemco plates (6.25 g/L Lemco, 6.25 g/L NaCl, 12.5 g/L Bacto peptone, 18.75 g/L Bacto agar) as described previously [29]. For *Mycobacterium smegmatis dut^D83N^* strain, we added hygromycin B (Calbiotech, El Cajon, CA, USA) at 100 μg/mL and gentamicin (Sigma, St.Louis, MO, US) at 10 μg/mL final concentration, respectively.

### 2.2. dUTPase Expression and Purification

The recombinant *Mycobacterium tuberculosis* dUTPase with an N-terminal 6xHis-tag [30] was expressed in the *Escherichia coli* strain BL21(DE3) (pLysS) in LB medium. The protein was overexpressed upon induction with 1 mM isopropyl-β-D-thiogalactopyranoside (IPTG) at OD_600_ = 0.6 for 3 h at 37 °C. The pellets were lysed in a buffer containing 50 mM TRIS pH 7.5, 5 mM benzamidine, 0.1 mM PMSF, 2.5 mg lysozyme, and a half tablet of EDTA-free protease inhibitor (Roche Diagnostics, Rotkreuz, Switzerland). The suspension was sonicated 3 × 1 min on ice and centrifuged at 15,550 g for 30 min at 4 °C. The supernatant was loaded on Novagen Ni-NTA column (Merck KGaA, Darmstadt, Germany) and purified according to the Novagen protocol. Salt-wash was followed by step-wash in 50 mM Tris–HCl, pH 7.5, buffer containing 30 mM NaCl, and 50 mM imidazole. dUTPase was eluted in 0.5 M imidazole, pH 7.5, and was subsequently dialyzed into a buffer containing 20 mM HEPES pH 7.5, 100 mM NaCl, and 5 mM MgCl_2_. Protein quality was assessed by SDS-PAGE; protein concentration in monomers was determined by UV spectrometry (λ_280_ = 2980 M^−1^ cm^−1^).

### 2.3. Dcd:dut Expression and Purification

The *Mycobacterium tuberculosis* pTBdcd7 Dcd:dut expression plasmid [31] was a kind gift from Martin Willemoes, University of Copenhagen, Denmark. The Dcd:dut was expressed in the *Escherichia coli* (*E. coli*) BL21(DE3)pLysS strain in LB medium. The protein was overexpressed upon induction with 0.5 mM IPTG at OD_600_ = 0.6 for 3 h at 37 °C. The pellets were lysed in a buffer containing 50 mM TRIS pH 7.5, 5 mM benzamidine, 0.1 mM PMSF, 2.5 mg lysozyme, and a half tablet of EDTA-free protease inhibitor (Roche). The suspension was sonicated 3 × 1 min on ice and centrifuged at 15,550× *g* for 30 min at 4 °C. The supernatant was purified on a Pierce Strong Anion Exchange Spin Column (Thermo Fisher Scientific, Walthman, MA, USA) according to the manufacturer’s protocol. Briefly, the column was equilibrated with the purification buffer (50 mM TRIS pH 7.5), and the supernatant was loaded to the column and centrifuged at 4000 rpm for 5 min. The column was then washed with the purification buffer two times. The samples were eluted with increasing salt concentration applied in the purification buffer (from 0 to 1 M NaCl in 100 mM steps and 1 mL volume for each fraction). We analyzed the protein fractions by SDS-PAGE and continued the purification with the most concentrated Dcd:dut fraction. For additional purification, we used gel filtration on a Superdex 75 column (GE Healthcare, Chicago, IL, USA) using an AKTA Explorer purifier. We analyzed the protein purity on SDS-PAGE and measured the concentration using UV absorbance in Nanodrop (λ_280_ = 11,460 M^−1^ cm^−1^). 

### 2.4. Measuring dCTP Deaminase Activity

The dCTP deaminase activity of the Dcd:dut enzyme was measured in a continuous spectrophotometric assay using the difference in the molar extinction coefficients between deoxycytidine and deoxyuridine (Δε_286_ = 3240 M^−1^ cm^−1^). The absorbance was recorded at 286 nm in a Specord 200 (Analytic Jena, Jena, Germany) spectrophotometer using 10 mm path-length quartz cuvettes thermostated at 20 °C. The assay was buffered with 20 mM HEPES pH 7.5, 100 mM NaCl, and 5 mM MgCl_2_. We added the 5-methyl-dCTP or 5-hydroxymethyl-dCTP into the Dcd:dut containing premix. After 30 min, dCTP was also added to the reaction mix.

### 2.5. dNTP Pool Extraction

Cells were grown until the culture reached the mid-exponential phase OD_600_ = 0.6. The total CFU count was determined for each culture used for dNTP extraction. Cell cultures were centrifuged (20 min, 3600× *g*, 4 °C), then cell pellets were resuspended in precooled 0.5 mL 60% methanol. After overnight incubation at −20 °C, the samples were boiled for 5 min at 95 °C, and the cell debris was removed by centrifugation (20 min, 16,000× *g*, 4 °C). The methanolic supernatant containing the soluble dNTP fraction was vacuum-dried (Eppendorf, Hamburg, Germany) at 45 °C. dNTPs were dissolved in 50 µL nuclease-free water and stored at −20 °C until use.

### 2.6. dNTP Quantification

We applied similar reaction conditions for the dNTP incorporation assay as described previously [2]. In detail, the 10 μL reaction mixture contained 2.5 pmol template (DNA sequences of the templates specific for each dNTP can be found in Supplementary Table S1); 2.75 pmol primer; a dNTP mix at 50 µM final concentration missing the dNTP to be quantified (it comes from the sample); 1.25 µM EvaGreen; 20 U/mL Q5^®^ High-Fidelity DNA Polymerase (New England BioLabs, Ipswich, MA, USA) for dTTP and dATP measurements, and 10 U/mL for dCTP and dGTP measurements; and 1x reaction buffer provided with the polymerase. For measuring dUTP+dTTP, we replaced the Q5 polymerase with the Q5U^®^ Hot Start High-Fidelity DNA Polymerase (New England BioLabs, Ipswich, MA, USA) at 20 U/mL final concentration in 1x Q5U reaction buffer. For measuring dTTP only, we included 4 µM *Mycobacterium tuberculosis* dUTPase in the reaction mix to digest off the dUTP content of the sample. For the selective elimination of dCTP, we incubated the samples with 0.6 µM *Mycobacterium tuberculosis* Dcd:dut at 37 °C for 1 h. We followed dNTP incorporation by the fluorescent signal of EvaGreen incorporation into the newly synthesized double-stranded DNA in a CFX96 Touch™ Real-Time PCR Detection System (Bio-Rad, Hercules, California, USA). The thermal profile was the following: 95 °C for 10 s, 75 °C for 1 s (data acquisition), 66 °C for 50 min (data acquisition every 5 min), and 75 °C for 5 s (data acquisition). We used FrameStar^®^ 96-Well Skirted PCR Plates with black wire and white wells sealed with adhesive PCR films. A dNTP calibration series established a quantitative correlation between nucleotide incorporation and the measured fluorescence changes. To record calibration curves, the reaction was supplied with 100–1000 fmol dNTP standards. The concentration of each stock of the standard dNTPs, templates, and primers were determined by measuring the absorbance at 260 nm. Oligonucleotides were from Sigma (standard purification); dNTP standards were from New England Biolabs.

### 2.7. Statistical Analysis

A two-way t-probe was used to test if dNTP concentrations measured by the fluorescence method for noncanonical dNTPs were different from the earlier published data measured by radioactive dNTP incorporation-based measurements. The obtained significance levels are specified in the legend of the relevant figure. Calibration curves were evaluated using regression coefficients (R^2^). The limit of detection (LOD) was determined by calculating the calibration line offset + 3 × SD F(low calibration points), where the offset of the calibration line and the standard deviation of the F values for parallel measurements were considered at low dNTP concentration. The limit of quantification (LOQ) was determined by calculating the calibration line offset + 5 × SD F(low calibration points), where the offset of the calibration line and the standard deviation of the F values for parallel measurements were considered at low dNTP concentration. Interassay coefficient of variation (CV%) were calculated by using means and standard deviation from two independent experiments performed on different days. The intraassay CV% were calculated using means and SD from two identical yet independent assays performed the same day.

## 3. Results

### 3.1. Choosing the Suitable Fluorescent Method for Non-Canonical dNTP Determination and Establishment of the dUTP Measurement Technique

**Figure 1:**
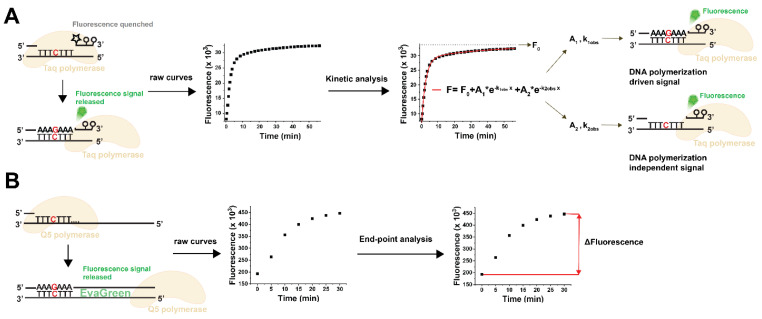
Comparison of the fluorescent dNTP incorporation methods. (**A**) Scheme of the TaqMan probe-based method using kinetic analysis [1]. Star represents the FAM fluorophore, while circles represent the quenchers on the probe. The schematic view shows a dGTP measurement; the red C represents the detection site for dGTP. Raw reaction curves are fitted with $F=F_{0}{+A}_{1}\times e^{-k_{1obs}x}+A_{2}\times e^{-k_{2obs}x}$ equation, where *F* is the observed fluorescence; *x* is the variable (time); *A*_1_ and *A*_2_ are fluorescence amplitudes; *k*_1*obs*_ and *k*_2*obs*_ are the rate constants of the observable fluorescence phases; and *F*_0_ is the y offset. The *A*_1_ and *k*_1*obs*_ parameters relate to the DNA-polymerization-dependent signal increase, while the *A*_2_ and *k*_2*obs*_ parameters are related to the DNA-polymerization-independent exonuclease activity of the polymerase. The *A*_1_ parameter is proportional to the incorporation of limiting dNTPs and is used for calibration and samples measurement. The kinetic analysis is performed by the dedicated and streamlined NucleoTidy software (http://nucleotidy.enzim.ttk.mta.hu/). (**B**) Scheme of the method based on the quantitative determination of double-stranded DNA [2]. The method uses long DNA templates suitable for EvaGreen detection if complemented by the synthesized complimentary strand. The schematic view shows a dGTP measurement; the red C represents the detection site for dGTP. The readout is the total fluorescence signal increase which is proportional to the limiting dNTP incorporated, and, therefore, Δ*F* is used for calibration and samples measurement.

The pioneering fluorescent assay used a TaqMan-like probe for fluorescence detection [26]. We further improved this method to eliminate non-specific disturbances by implementing a kinetic evaluation of the dNTP incorporation time courses (Figure 1A) [1]. Although our modified assay performed well in biological samples for the canonical dNTP measurements, we could not apply this method to measure noncanonical dNTPs. At first, we tested the TaqMan probe-based method for dUTP measurement [1]. We used various predetermined dUTP:dTTP ratios in the reaction mixtures, but we could not entirely recover the dUTP below a 1:2 dUTP:dTTP ratio. This ratio is considerably higher than what is typically observed in biological samples. The TaqMan probe-based assay did not work for dUTP due to the kinetic difference in dTTP and dUTP incorporation, dUTP incorporation being significantly slower than dTTP incorporation under the same conditions (cf. Figure 2A in Szabó et al., 2020 [1]). Since the different kinetics of dUTP and dTTP incorporation hinders the unequivocal assignment of the kinetic readout to dUTP or dTTP, we needed an end-point fluorescent method to overcome the problem.

**Figure 2:**
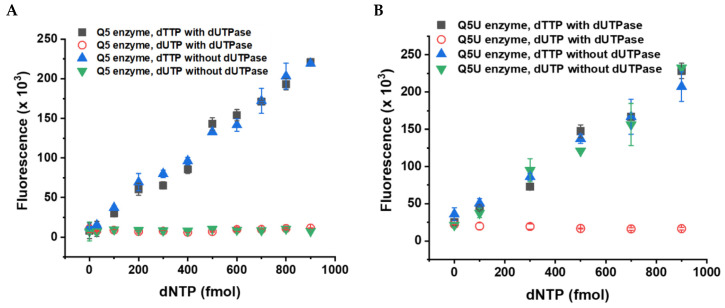
Difference between the Q5 and Q5U enzymes in accepting dUTP as a substrate. (**A**) The Q5 enzyme does not incorporate dUTP into the DNA; therefore, the presence of dUTPase enzyme does not influence the dTTP measurement. (**B**) The Q5U enzyme incorporates dUTP into the DNA as efficiently as dTTP against the same template and in the same conditions (green triangles). All data points represent the mean and standard error of two independent experiments with two technical replicates (four measurements in total).

In parallel with our kinetic method, another fluorescent method was published that uses a simpler toolkit than the TaqMan-like assay. This approach is based on the incorporation of the dNTP species to be quantified to a specific site near the 3’ end of the template (C in red in Figure 1B). The resulting quantity of double-stranded DNA molecules directly correlates with the concentration of the target dNTP species in the sample. Signal detection simply relies on the standard double-stranded DNA detection of the qPCR assays using the EvaGreen dye (Figure 1B). This detection method relies on DNA templates significantly longer than those in the TaqMan-like assay (cf. Figure 1A,B). Additionally, an exceptionally accurate DNA polymerase is required, as the incorporation of a single building block into an extended DNA strand establishes a proportional relationship between the dNTP being measured and the synthesized DNA strand (Figure 1B) [2]. This method offers the finest sensitivity among fluorescent dNTP incorporation assays, and its sensitivity compares to that of HPLC-MS methods. The assay developed by Purhonen et al. is a real end-point method [2], and, therefore, we tried this assay for dUTP measurement. Unexpectedly, we found that the Q5 DNA polymerase optimized for this assay does not incorporate dUTP into the nascent DNA strand (Figure 2A). Therefore, we assayed another enzyme claimed to process uracil-containing templates, the Q5U DNA polymerase. We found that Q5U is not limited to handling templates containing uracil; it is equally proficient at integrating dUTP against the adenine base present in the template (Figure 2B). Therefore, the measurement of samples containing both dTTP and dUTP will report the sum of the amount of dTTP and dUTP in the sample. To distinguish between these nucleotides, we employed the dUTPase enzyme to specifically digest dUTP to dUMP, leaving dTTP intact (Figure 2B). For assessing the dUTP content in biological samples, we propose a two-step procedure. First, we quantitate both dTTP and dUTP simultaneously by utilizing the Q5U polymerase. Then, we separately measure the dTTP content in the same sample after subjecting it to dUTPase digestion. The difference between these two measurements will represent the dUTP quantity in the sample. Details regarding the performance of the measurement are presented in Table 1.

### 3.2. Validation of the Fluorescent dUTP Measurement Technique

**Figure 3:**
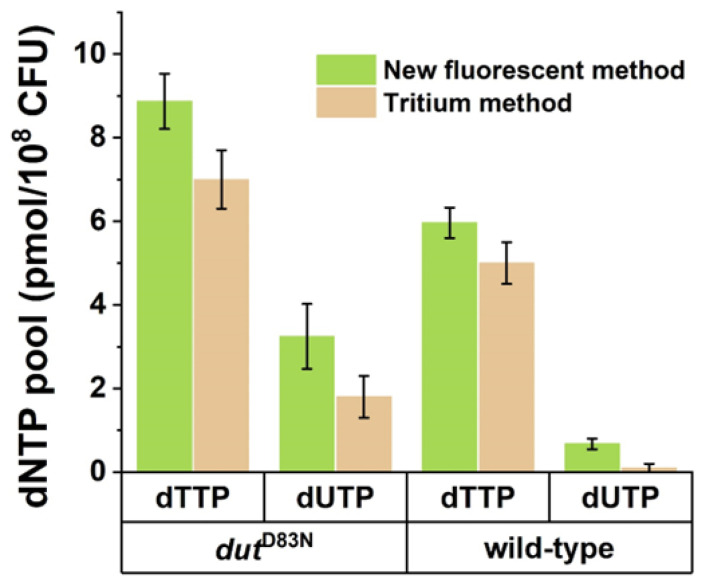
Comparison of the fluorescent and radioactive isotope (tritium) incorporation-based methods for dUTP quantification. dTTP and dUTP quantification was carried out on samples from wild-type and dUTPase inactive (*dut^D83N^*) *Mycobacterium smegmatis* strains. Data obtained by radioactive detection were extracted from Hirmondó et al., 2017 [22,25]. dNTP quantities measured from extracts of 10^8^ viable cells are shown. Differences were assessed by paired-sample *t*-test resulting in the following significance levels: mutant dTTP, *p* = 0.0834; mutant dUTP, *p* = 0.0621; wild-type dTTP, *p* = 0.1994; and wild-type dUTP, *p* = 0.0125. Data points represent the mean and standard deviation of three independent biological samples. For technical parallels, four different dilutions for each biological sample were measured.

We previously determined the dUTP content of *Mycobacterium smegmatis* wild-type and *dut^D83N^* mutant strains using a radioactive dNTP incorporation measurement method [25]. To test the performance of the newly developed fluorescent protocol on biological samples, we subjected samples from these two mycobacterial strains to dUTP measurement using the Q5U polymerase. We found that applying our newly established protocol resulted in the same pattern and same dNTP concentrations within the margins of error of the two methods in the measurable range (Figure 3). The observed differences between the fluorescent and radioactive methods were nonsignificant in the paired-sample *t*-test with the exception of the wild-type dUTP samples. In this case, the dUTP content of the sample was at the lower detection limit of the radioactive assay, with 100% relative error. This makes this result less reliable than the one yielded by our new, fluorescent method. It must be noted that each type of biological sample may need an optimization of the reaction conditions due to the matrix effects experienced using both the Purhonen et al. [2] and the Szabó et al. [1] methods.

### 3.3. Establishment of the 5-Methyl-dCTP and the 5-Hydroxymethyl-dCTP Measurement Technique

**Figure 4:**
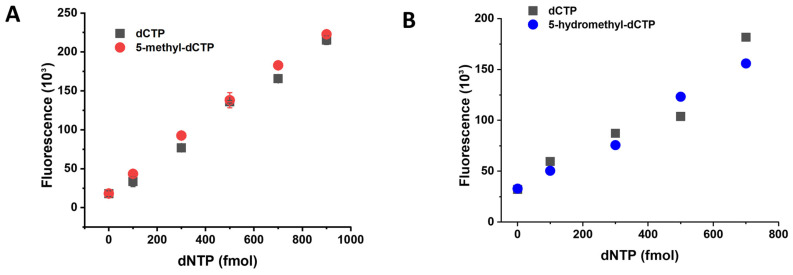
The 5-methyl-dCTP and 5-hydroxymethyl-dCTP are incorporated into DNA as efficiently as dCTP by the Q5 enzyme. (**A**) Comparison of the incorporation of 5-methyl-dCTP and dCTP into the same template and in the same conditions. (**B**) Comparison of the incorporation of 5-hydroxymethyl-dCTP and dCTP into the same template and in the same conditions. All data points represent the mean and standard error of two independent experiments with two technical replicates.

We further aimed to extend the fluorescent dNTP measurement capability with the quantification of 5-methyl-dCTP and 5-hydroxymethyl-dCTP. We tested the dNTP measurement assay conditions of the dCTP reaction for 5-methyl-dCTP and 5-hydroxymethyl-dCTP using the Q5 DNA polymerase (Figure 4A,B, Table 1). As shown in Figure 4A,B, both 5-methyl-dCTP and 5-hydroxymethyl-dCTP are incorporated into DNA by the Q5 polymerase similarly to dCTP. To differentiate the noncanonical dCTP forms from the canonical dCTP, we first considered using the mycobacterial MutT2 enzyme as it was shown to be the most active on 5-methyl-dCTP [19]. Unfortunately, this enzyme also digests other dNTPs, including dCTP, making it inapplicable in this assay [19].

**Figure 5:**
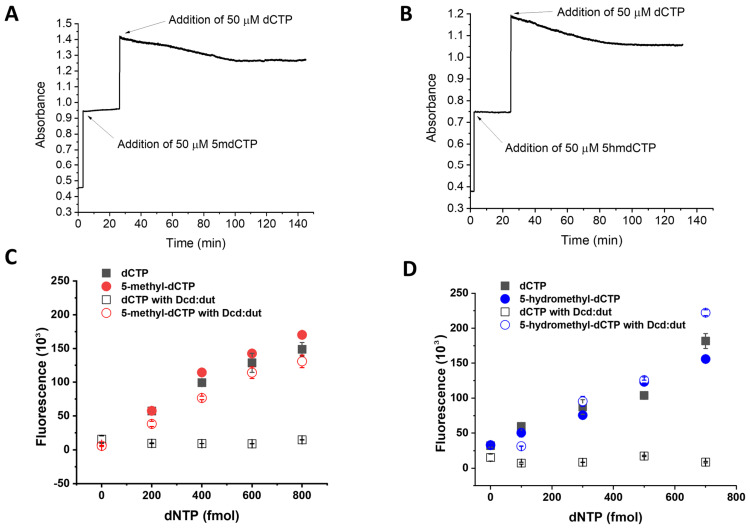
The Dcd:dut enzyme digests dCTP but not 5-methyl-dCTP (5mdCTP) or 5-hydroxymethyl-dCTP (5hmdCTP). (**A**) Time course of 5-methyl-dCTP and dCTP hydrolysis by Dcd:dut. At t = 1 min, 5-methyl-dCTP was added to the reaction mix containing 0.21 μM Dcd:dut. During the 30 min incubation, no reaction (i.e., decrease in absorbance) was detectable. The observed time-dependent absorbance decrease upon the addition of dCTP into the same enzymatic reaction mixture indicates that the assay was functional. The plateau starting at approximately 100 min indicates the depletion of dCTP. (**B**) Same experiment as in panel A using 5-hydroxymethyl-dCTP instead of 5-methyl-dCTP. (**C**) Selective quantification of 5-methyl-dCTP using Dcd:dut enzyme to eliminate dCTP from the sample. (**D**) Selective quantification of 5-hydroxymethyl-dCTP using Dcd:dut enzyme to eliminate dCTP from the sample. All data points represent the mean and standard error of two independent experiments with two technical replicates.

The *Mycobacterium tuberculosis* Dcd:dut enzyme was previously shown to convert dCTP into dUMP [25,31]. Its activity on 5-methyl-dCTP and 5-hydroxymethyl-dCTP, however, was not established. Based on the fact that the inhibitory effect of dTTP on this enzyme is mainly achieved by the methyl-group on the C_5_ of the pyrimidine ring, we hypothesized that 5-methyl-dCTP and 5-hydroxymethyl-dCTP are weak substrates for Dcd:dut digestion. We, therefore, produced the recombinant *Mycobacterium tuberculosis* Dcd:dut enzyme in an active form. We found that none of the noncanonical dCTPs were hydrolyzed by the *Mycobacterium tuberculosis* Dcd:dut, while dCTP was readily digested in the same reaction mixture (Figure 5A,B). Based on this result, the *Mycobacterium tuberculosis* Dcd:dut is suitable for selective dCTP elimination. Indeed, when incorporating Dcd:dut to the dNTP quantification method, we could distinguish between dCTP and modified dCTP analogues (Figure 5C,D). Taking advantage of this novel finding, we implemented a two-step method for 5-methyl-dCTP and 5-hydroxymethyl-dCTP measurements similarly to the dUTP measurement. In simultaneous assays, canonical and noncanonical dCTPs are quantified as a combined measurement, while the content of 5-methyl-dCTP and 5-hydroxymethyl-dCTP in the same sample is determined separately following enzymatic digestion with Dcd:dut. The difference between the two measurements will yield the noncanonical dCTP content.

## 4. Discussion

Organisms possess a diverse array of enzymes designed to remove noncanonical dNTPs from their dNTP pools, and this serves a crucial purpose. The existence of noncanonical or modified nucleobases in DNA can either lead to errors or serve as specific signals. In both scenarios, the unregulated incorporation of these noncanonical dNTPs could have significant consequences for the fate of the cell. The majority of DNA polymerases do not have mechanisms to differentiate between canonical and noncanonical dNTPs. Consequently, the control of dNTP incorporation can only be enforced prior to replication by ensuring dNTP pool sanitation, or post-replication through DNA repair mechanisms. The noncanonical content of the dNTP pool is, therefore, also relevant for the correct interpretation of DNA sequencing results, with a particular focus on mutational patterns and DNA repair deficiencies. In this paper, we report the quantitative determination of noncanonical dNTPs that, if incorporated by error, may confuse physiological signals to be generated in DNA in a controlled manner. These novel quantification protocols will be useful for all those who conduct studies in biomedically relevant fields discussed below.

Uracil in DNA drives somatic hypermutation in functional antibody genes which creates the vast antibody diversity required for the detection and response to microorganisms [32]. However, dUTP incorporation from the dNTP pool to other genomic regions may be detrimental [14]. There is an increasing interest in one-carbon metabolism and in the contribution of dUTP to it, as well as in other potential roles of dUTP in physiological processes [17,33,34,35,36].

The methylation of cytosine in DNA is an important epigenetic signal involved in the differential control of gene expression. Methylation occurs in the C5 position of cytosine by DNA (cytosine-5) methyltransferase in its regulated pathway [37]. However, 5-methyl-dCTP is an excellent analogue of dCTP and can be incorporated into DNA as shown in Figure 4, and as was previously demonstrated by others using *E. coli* DNA polymerases [38,39]. The intracellular source of 5-methyl-dCTP may primarily come from the degradation of methylated DNA.

Previous results also demonstrated that mammalian cells can uptake and incorporate labelled 5-methyl-deoxycytidine during replication, which results in a measurable increase in DNA methylation levels [15,40]. These observations imply that these cells express at least one kinase that phosphorylates 5-methyl-deoxycytidine to 5-methyl-dCTP. Although such mammalian kinases have not yet been identified, phage kinases that can phosphorylate 5-methyl-dCMP [39] and 5-hydroxymethyl-dCMP [41] have been reported. Further indirect evidence of the relevance of 5-methyl-dCTP in the dNTP pool is that dNTP pool sanitation enzymes with highly specific 5-methyl-dCTP hydrolase activity are present in both *E. coli* (Orf135 [42]) and mammals (RS21-C6 in mice [43]; DCTPP1 in human [15]).

In conclusion, readily available techniques for quantifying noncanonical dNTPs are relevant and necessary in order to expand our understanding about the physiological composition of the cellular DNA precursor pools. More data on the noncanonical content of the dNTP pools will advance our understanding of the regulation of DNA metabolism and repair processes. Furthermore, it will greatly facilitate the interpretation of the extensive data generated by next-generation sequencing experiments.

## Figures and Tables

**Table 1:** Analysis of the assay performance.

	Tested Range (pmol)	R^2^	LOD (pmol)	LOQ (pmol)	Accuracy Low (%)	Accuracy High (%)	Inter-Assay CV%	Intra-Assay CV%
dUTP	30–1000	0.95 ± 0.01	66 ± 35	110 ± 59	108 ± 13	102 ± 4	9.65 ± 5.33	14.96 ± 1.17
hmdCTP/5m-dCTP	30–1000	0.96 ± 0.04	37 ± 1	61 ± 1	99 ± 11	101 ± 1	8.82 ± 12.11	13.46 ± 12.45

R^2^: regression coefficient; LOD: limit of detection; LOQ: limit of quantification; CV: coefficient of variation.

## Data Availability

The data presented in this study are contained within the article.

## Supplementary Materials

The following supporting information can be downloaded at: https://www.mdpi.com/article/10.3390/biom13121801/s1, Table S1: Oligonucleotides used in the present study.
