# Supplementary material for: Using Selective Enzymes to Measure Noncanonical DNA Building Blocks: dUTP, 5-Methyl-dCTP, and 5-Hydroxymethyl-dCTP
Source: Biomolecules. 2023 Dec 15;13(12):1801. doi: 10.3390/biom13121801 (PMC10742078; doi:10.3390/biom13121801)
Supplement: Supplementary file 1 [file biomolecules-13-01801-s001.zip › biomolecules-2738377-supplementary.pdf]

Supplementary Materials

Supplementary Table S1: Oligonucleotides used in the present study

Oligo name	5′-3′ sequence
Detection primer	CCGCCTCCACCGCC
dTTP-template-197nt	TCGCTCGCTCTTGCCTCGGTCCTCGCTCGCTCTTGCCTCGGTCCTCGCTCGC<br>TCTTGCCTCGGTCCTCGCTCGCTCTTGCCTCGGTCCTCGCTCGCTCTTGCCTC<br>GGTCCTCGCTCGCTCTTGCCTCGGTCCTCGCTCGCTCTTGCCTCGGTCCTCGC<br>TCGCTCTTGCCTCGGTCCTTTATTT**GGCGGTGGAGGCGG**
dCTP-template-197nt	CCACTCACTCTTACCTCAATCCCCACTCACTCTTACCTCAATCCCCACTCACTC<br>TTACCTCAATCCCCACTCACTCTTACCTCAATCCCCACTCACTCTTACCTCAAT<br>CCCCACTCACTCTTACCTCAATCCCCACTCACTCTTACCTCAATCCCCACTCAC<br>TCTTACCTCAATCCTTTGTTT**GGCGGTGGAGGCGG**

The primer binding sites are shown in bold and the detection sites are marked red.
